# The association between physical activity and fear of falling among community-dwelling older women in China: the mediating role of physical fitness

**DOI:** 10.3389/fpubh.2023.1241668

**Published:** 2023-08-14

**Authors:** Shuang Wu, Guangkai Li, Beibei Shi, Hongli Ge, Qiang He

**Affiliations:** School of Physical Education, Shandong University, Jinan, China

**Keywords:** older women, physical activity, fear of falling, physical fitness, mediation analysis

## Abstract

**Background:**

This study aimed to explore the association between physical activity (PA) and fear of falling (FOF) and to determine whether this relationship was mediated by physical fitness (PF) in community-dwelling older women.

**Methods:**

For this cross-sectional study, a total of 1,108 older women were recruited. Moderate-to-vigorous physical activity (MVPA) and light physical activity (LPA) were objectively measured by accelerometers. Physical fitness indicators including body mass index (BMI), forced vital capacity, choice reaction time, grip strength, sit and reach, and five times sit-to-stand were measured. FOF was evaluated by the Chinese version of the activities-specific balance confidence scale. A stepwise linear regression model analysis was used for mediation analysis, and bootstrap analysis was used to verify the mediation effects.

**Result:**

The Pearson correlation coefficient results suggested that MVPA was significantly and negatively correlated with FOF while LPA was not correlated with FOF. Logistic regression analysis suggested a significant association between MVPA, BMI, forced vital capacity, choice reaction time, sit and reach, grip strength, five times sit-to-stand, and FOF. The mediation analysis showed a negative relationship between MVPA and FOF. BMI completely mediated the relationship between MVPA and FOF while sit and reach, five times sit-to-stand, and forced vital capacity partially mediated the relationship between MVPA and FOF.

**Conclusion:**

Accumulation of more daily MVPA was associated with reducing the odds of FOF in community-dwelling older women. PF indicators fully or partially mediate the relationship between MVPA and FOF. Therefore, more intervention efforts should focus on the promotion of MVPA to improve PF and thus reduce FOF among older women.

## Introduction

Falls represent a major public health threat as falls are the leading cause of injury-related morbidity and mortality among older adults (1, 2). Consequences of falls can be severe, including fractures, increased risk of disability, premature nursing home admissions, and substantial reduction in quality of life. It was suggested that approximately one in three community-dwelling older adults experience at least one fall each year (3). Moreover, this figure increases to two in three for those who have a history of fall or are afraid of falling (4).

Fear of falling (FOF) is considered low perceived self-efficacy in avoiding falls during essential, non-hazardous activities of daily living (5). FOF is very common among community-dwelling older adults with a prevalence ranging from 21 to 85% (6). An earlier review suggested that older adults with falling experience had a prevalence of FOF ranging from 29 to 92% while older adults who had not fallen had a prevalence ranging from 12 to 65% (7). There were a series of risk factors for FOF including but not limited to old age, female sex, worse physical performance, fall history, poor self-rated health, cognitive impairment, depressive symptoms, and living alone (8). The consequence of FOF includes more frequent falls (9), a decline in physical and mental performance, and a reduction in quality of life (10) and social activity (11). Apparently, being female is one of the main risk factors robustly associated with developing FOF (12). Therefore, more attention should be paid to older women with FOF.

Recent evidence suggested that a sedentary lifestyle is a potential risk factor for FOF (13), while an increasing physical activity (PA) level probably is a promising strategy to protect against FOF (14). The health benefits of PA had been widely accepted including but not limited to reducing the risk of cardiovascular disease, diabetes, and all-cause mortality (15, 16). Several studies have demonstrated that PA is associated with FOF in the older population; however, the results and conclusion were not always consistent (17–19). In addition, in most cases, the influence of FOF on PA was examined while the influence of PA on FOF was scarcely studied.

Physical fitness (PF) is an important health marker for children and adults (20, 21). Poor PF is associated with the prevalence of various chronic conditions (22, 23). Studies consistently demonstrated that older adults with FOF presented worse physical performance (24–27). On the contrary, older adults with high PF performance presented lower FOF level. Thus, it is reasonable to assume that the improvement in FOF is due, at least in part, to a better PF. Thus, it is necessary to clarify whether a low level of FOF among older people is due to better PF. At the same time, a positive association between PA and PF was supported by previous findings which linked PA with lower body mass index (BMI), better aerobic endurance and strength (28), and better balance capacity in older adults (29). These findings suggested that PF can act as an intermediate variable between PA and FOF. A deeper understanding of the potential mediating effects of PF in the relationship between PA and FOF is required.

Therefore, this study aimed to analyze whether PA measures including LPA and MVPA is associated with FOF in a sample of community-dwelling older women and the potential mediating role of PF in these associations was determined.

## Methods

### Covariate variables

Sociodemographic data were collected by self-report, including age, education level (primary school and below, middle school, and undergraduate and above), living alone (yes, no), current drinkers (yes, no), fall experience (no falls, ≥1 falls), type of medications taken (< 5, ≥5), and diagnosed chronic disease (no, yes ≥1). The nutritional and cognitive statuses were assessed using short-form mini-nutritional assessment (MNA-SF) (30) and mini-mental state examination (MMSE) (31), respectively. Muscle mass was measured by bioimpedance analysis (BIA) using the Tanita MC-180 (Bailida Co., Tokyo, Japan) (32). The balance stable coefficient was assessed using the Super Balance III Static Balance Test System (Acmeway, Beijing, China) (33).

### Study design and participants

This study used a cross-sectional design with data from the baseline survey of Physical Activity and Health in Older Women Study (PAHIOWS) (34). Convenience sampling was used to recruit participants from urban communities in Yantai, Shandong province in China. The time span was from March 2021 to June 2021. Convenience sampling is a specific type of non-probabilistic sampling method that relies on data collection from populations who are conveniently available to participate in the study, and it is most frequently used in quantitative studies (35). A total of 1, 370 older women were recruited, and participants meeting the following criteria were finally included: (1) participants aged 60–70 years, (2) participants who have no cognitive impairment and can communicate normally, (3) participants who have no physical disability that might impede normal physical activity, and (4) participants who are willing to sign the informed consent form. Overall, 1, 108 participants were included in the data analysis. This study was approved by the Ethics Committee of the School of Nursing and Rehabilitation, Shandong University, China (2021-R-001). We measured and collected data related to physical activity, fear of falling, and physical fitness of eligible subjects.

### Physical activity

The participants were required to wear the Actigraph WGT3X-BT (Actigraph, Pensacola, FL, USA) accelerometer on their left waist for 7 consecutive days to objectively measure the LPA and MVPA (36). As the number of accelerometer devices is limited, participants were invited to participate in the physical activity measurement in batches. In general, every day, 20–40 participants were invited to our test center to complete the fitness test and relevant questionnaire survey and then wear the accelerometer. Therefore, the physical activity measurement can be deemed and implemented within the same time frame. The sampling frequency of the original accelerometer data is 30 times per second, and the original data are converted into 60-s epoch length motion counts for further analysis. Non-wearing time was defined as at least 90 consecutive min of 0 counts, with an allowance of a maximum of 2 min of counts between 0 and 100 counts per minute (CPM) (37). Valid data are defined as the wearing time being more than or equal to 10 h per day and the wearing days being more than or equal to 4 days (38). Cutoff points defined by Freedson were used to classify sedentary time (SB), LPA, and MVPA as follows: SB (0 to 99 CPM), LPA (100 to 1951 CPM), and MVPA (≥1952 CPM) (39).

### Fear of falling

The Chinese version of the activity-specific balance confidence (ABC) scale was used to evaluate the FOF. ABC is a mature and structured scale that is more suitable for older adults with certain physical activities (40). A score of 88 was taken as the cutoff point, and a high FOF was judged as an ABC score lower than 88 (34). During the assessment, participants were asked to report their level of confidence in specific physical activities (walking on the icy road, walking up and down the escalator without holding the handrail, etc.). The confidence ranges from 0% (no confidence) to 100% (full confidence). If participants had no physical activity in reality, they were asked to imagine themselves in this specific physical activity and give corresponding confidence levels.

### Physical fitness

The physical fitness test includes six standardized tests, namely BMI, forced vital capacity, sit and reach, grip strength, five times sit-to-stand, and choice reaction time. For the physical fitness test, we used the fitness management system (Acmeway company, Beijing, China).

The obesity degree was evaluated by body mass index (BMI), which was obtained by dividing the weight (kg) of a subject by the square of height (m) (41).

The forced vital capacity (FVC) is a readily available objective measurement of lung volume and respiratory muscle function (42). The subjects breathed as deeply as possible and then exhaled air from the lung. The greatest among of air that can be forced from the lung can be recorded by a spirometer (ml).

The coordination and rapid reaction ability of human nervous and muscular systems were evaluated by choice reaction time (43). The subjects were instructed to press the “start key” and wait for the signal to be sent. When any “signal key” sends a signal (light), the subjects press the corresponding key as quickly as possible and then return to the “start key” waiting for the next signal. Five signal responses should be answered in each test, and the time was recorded (seconds).

The upper limb muscle strength was evaluated by the grip strength test (41). A manual dynamometer was used to assess grip strength. The test was performed in a standing position with the arm naturally down on both the right and left sides. During the test, the upper and lower handles were tightly held with maximum force, and the measured strength was recorded in kilograms.

The flexibility of the lower limb and the trunk was evaluated by sit and reach test (44). The subjects sat on the floor with their head, back, and hips against a wall, knees straight, heels together, and feet positioned flat against a board of the sit and reach the box. The subjects were required to extend their arms with palms down and bend forward slowly, pushing the vernier forward with the fingertips of the middle fingers of both hands as far forward as possible while keeping the knees extended.

The dynamic balance and lower limb muscle strength were evaluated through five times sit-to-stand test (45, 46). The subjects were required to sit on a chair of height between 43 and 46 cm, with arms crossed over their chest. During the test, they should stand up and then return to the sitting position as fast as possible, and the time to finish five repetitions was recorded (seconds).

All tests are performed twice, taking the better one. Moreover, all testers were provided with a manual describing the standardized procedure, demonstrations, and verbal cure for participants.

### Statistical analyses

Descriptive data were presented as means and standard deviations (SDs) or as numbers and percentages (%). Differences between the low FOF group and the high FOF group were assessed using the Mann–Whitney test, Fisher's exact test, chi-square test, and trend chi-square test, depending on the type of variables. Pearson's correlation coefficients and logistic regression model were used to examine the relationships between FOF, PA, and PF. The stepwise linear regression model is used to determine whether the association between PA and FOF was mediated by PF. Bootstrap analysis was used based on sample sampling by 5000 times to observe whether the 95% confidence interval of the indirect effect crosses 0 to further judge whether there is a mediating effect. The mediating effect needs to meet the following conditions: (1) The total effect c is significant that PA could significantly affect the FOF (path c); (2) the indirect effect a × b is significant that PA could significantly affect the PF and PF could significantly affect the FOF.

Before running these models, the accelerometer-measured MVPA and LPA were rescaled so that a one-unit increase reflected 30 min/day. In addition, we mainly adjusted the age, wearing time, education level, living alone, drinking, drug use, chronic diseases, MMSE score, muscle mass, MNA score, fall experience, and balance stability coefficient in Model 1. Model 2a adjusted the sedentary time based on Model 1 (stepwise linear regression model and bootstrap analysis used Model 2a). While Model 2b adjusted MVPA time based on Model 1, Model 2c adjusted MVPA time and sedentary time based on Model 1. The variance inflation factor (VIF) for all variables was calculated to detect collinearity. The VIF is considered acceptable when it is < 5 (47). All analyses were conducted using STATA MP software version 17.0 (StataCorp, Texas, USA). The statistical significance was set at a *p*-value of < 0.05 in two-sided tests.

## Results

Table 1 shows the basic characteristics of the participants. A total of 1, 108 older women were included in the final analysis, with an average age of 64.9 ± 2.8 years. A total of 210 older women and 898 older women were identified as high FOF and low FOF in the total sample, respectively. More than a quarter of participants had fallen once or more. The participants spent an average of 548.2 min on sedentary behavior and 32.7 min and 307.5 min per day on MVPA and LPA, respectively. There were significant statistical differences in age, ABC score, education level, fall experience, MVPA, sedentary time, BMI, forced vital capacity, choice reaction time, sit and reach, grip strength, and five times sit-to-stand between the high and low FOF group. No significant inter-group differences were found in other indicators.

**Table 1:** Characteristics of participants.

**Variables**	**FOF group score**			
	**Total (*****n** =* **1,108)**	**Low FOF (*****n** =* **898)**	**High FOF (*****n** =* **210)**	***p***
Age, years	64.9 (2.8)	64.8 (2.8)	65.4 (2.8)	0.005
Living alone (%)	125 (11.3)	107 (11.9)	18 (8.5)	0.147
Education (n, %)				0.012
Primary school or lower	133 (12.0)	96 (10.6)	37 (15.1)	
Middle school	774 (69.8)	634 (70.1)	140 (65.7)	
Bachelor degree or higher	201 (18.1)	171 (19.0)	30 (14.9)	
Current drinker (%)	105 (9.5)	84 (9.3)	21 (9.8)	0.642
No. of medications taking ≥ 5 (%)	14 (1.3)	9 (1.0)	5 (2.3)	0.111
Diagnosed Chronic diseases (%)	720 (64.9)	574 (63.9)	146 (69.5)	0.121
MMSE score	26.7 (1.4)	26.7 (1.4)	26.5 (1.5)	0.060
MNA-SF	13.3 (1.1)	13.3 (1.0)	13.2 (1.3)	0.929
ABC score	92.9 (8.9)	96.4 (3.6)	77.1 (10.1)	< 0.001
Fall experience ≥ 1 (n, %)	315 (28.2)	230 (40.1)	85 (25.5)	< 0.001
Muscle mass (kg)	38.7 (2.8)	38.68 (2.8)	38.7 (3.0)	0.924
Balance stable coefficient	12.5 (1.9)	12.6 (1.9)	12.1 (2.1)	0.008
Wear time (min/day)	888.1 (118.4)	886.8 (115.7)	891.3 (128.9)	0.824
MVPA (min/day)	32.7 (19.3)	33.7 (18.9)	28.52 (20.4)	< 0.001
LPA (min/day)	307.5 (70.5)	308.7 (71.6)	302.2 (65.7)	0.440
SB (min/day)	548.3 (117.4)	544.9 (115.2)	563.0 (125.6)	< 0.001
BMI (kg/m^2^)	26.3 (3.4)	26.1 (3.2)	27.2 (3.9)	< 0.001
Forced vital capacity (ml)	2037.5 (537.9)	2065.3 (529.7)	1919.5 (557.4)	< 0.001
Choice reaction time (s)	0.6 (0.1)	0.6 (0.1)	0.7 (0.1)	< 0.001
Sit and reach (cm)	10.4 (9.2)	11.1 (8.9)	7.6 (9.8)	< 0.001
Grip strength (kg)	24.0 (5.0)	24.3 (5.0)	23 (4.5)	< 0.001
Five times sit-to-stand (s)	7.96 (2.1)	7.82 (2.0)	8.56 (2.2)	< 0.001

ABC, activities-specific balance confidence scale; MMSE, mini-mental state examination; MNA-SF, short-form mini-nutritional assessment; MVPA, moderate-to-vigorous physical activity; LPA, light-intensity physical activity; SB, sedentary behavior; BMI, body mass index.

Table 2 shows the Pearson correlation coefficients between PA, PF indicators, and FOF. It was suggested that the ABC score was positively associated with MVPA, forced vital capacity, sit and reach, and grip strength and negatively associated with choice reaction time, five times sit-to-stand, and BMI. Additionally, MVPA was positively associated with forced vital capacity and sit and reach and negatively associated with five times sit-to-stand and BMI. LPA was positively associated with sit and reach and negatively associated with five times sit-to-stand.

**Table 2:** Pearson's correlation coefficients between PA, PF, and FOF.

	**ABC score**	**MVPA**	**LPA**	**Forced vital capacity**	**Choice reaction time**	**Sit and reach**	**Grip strength**	**Five times sit-to-stand**	**BMI**
ABC score	**-**	0.12^**^	0.04	0.13^**^	−0.13^**^	0.21^**^	0.15^**^	−0.19^**^	−0.14^**^
MVPA		-	0.15^**^	0.10^**^	−0.07^*^	0.19^**^	0.04	−0.20^**^	−0.21^**^
LPA			-	0.00	0.05	0.15^**^	0.03	−0.07^*^	0.06
Forced vital capacity				-	−0.24^**^	0.11^**^	0.25^**^	−0.06	−0.14^**^
Choice reaction time					-	−0.08^**^	0.17^**^	0.24^**^	0.13^**^
Sit and reach						-	−0.18^**^	−0.24^**^	−0.17^**^
Grip strength							-	−0.11^**^	0.10^**^
Five times sit-to-stand								-	0.15^**^

MVPA, moderate-to-vigorous physical activity; LPA, light-intensity physical activity; BMI, body mass index. ^*^p < 0.05, ^**^p < 0.01.

According to the logistic regression analysis (Table 3), after adjusting all covariate variables in models 2a and 2b, the increase in daily MVPA per 30 min was significantly associated with FOF (OR = 0.72, 95% CI: 0.54–0.96, *p* = 0.023) while the increase in daily LPA per 30 min (OR = 0.97, 95% CI: 0.90–1.04, *p* = 0.35) was not correlated with FOF. Through model 2c, we found a significant association between FOF and BMI (OR = 1.12, 95% CI: 1.06–1.18, *p* < 0.000), forced vital capacity (OR = 0.96, 95% CI: 0.93–0.99, *p* = 0.003), choice reaction time (OR = 1.22, 95% CI: 1.06–1.41, *p* = 0.006), sit and reach (OR = 0.96, 95% CI: 0.95–0.98, *p* < 0.000), grip strength (OR = 0.94, 95% CI: 0.91–0.98, *p* = 0.001), and five times sit-to-stand (OR = 1.14, 95% CI: 1.06–1.23, *p* < 0.000), respectively.

**Table 3:** Logistic regression of the associations between PA, PF, and FOF.

**Variables**	**Odds ratio (95 % confidence interval)**			
	**Model 1**	***p***	**Model 2**	***p***
MVPA, per 30 min increased	0.67 (0.51, 0.86)	0.002	0.72 (0.54, 0.96)^a^	0.023
LPA, per 30 min increased	0.95 (0.89, 1.02)	0.188	0.97 (0.90, 1.04)^b^	0.350
BMI (kg/m^2^)	1.12 (1.07, 1.18)	< 0.000	1.12 (1.06, 1.18)^c^	< 0.000
Forced vital capacity (ml)	0.95 (0.93, 0.98)	< 0.000	0.96 (0.93, 0.99)^c^	0.003
Choice reaction time (s)	1.22 (1.06, 1.41)	0.005	1.22 (1.06, 1.41)^c^	0.006
Sit and reach (cm)	0.96 (0.95, 0.98)	< 0.000	0.97 (0.95, 0.98)^c^	< 0.000
Grip strength (kg)	0.94 (0.90, 0.97)	< 0.000	0.94 (0.91, 0.98)^c^	0.001
Five times sit-to-stand (s)	1.16 (1.08, 1.25)	< 0.000	1.14 (1.06, 1.23)^c^	< 0.000

Model 1, adjusted age, wearing time, education level, living alone, drinking, drug use, chronic diseases, MMSE score, muscle mass, MNA score, fall experience, balance stability coefficient; Model 2a, variables in Model 1+ daily sedentary time; Model 2b, variables in Model 1+ daily MVPA time; Model 2c, variables in Model 1+ daily MVPA time +daily sedentary time; BMI, body mass index.

As shown in Table 4, the total effect (c) suggested a significant positive relationship between MVPA and ABC score (β = 1.32, 95% CI: 0.43–2.20), but after adding mediator factors, only four significant indirect effects (a × b) were found, including forced vital capacity (β = 0.15, 95% CI: 0.02–0.27), sit and reach (β = 0.30, 95% CI: 0.10–0.50), five times sit-to-stand (β = 0.34, 95% CI: 0.12–0.56), and BMI (β = 0.51, 95% CI: 0.15–0.86), respectively.

**Table 4:** Mediation analysis results of PA, PF, and FOF.

**Mediator factors**	**PA (MVPA) → FOF (ABC score)**	**PA (MVPA) → PF**	**PA (MVPA) → PF → FOF (ABC score)**	Indirect effect: a^*^b	**Direct effect: c'**	**Total effect: c**	**Proportion**
Forced vital capacity	c: 1.32 (0.43, 2.20)^**^	a: 87.46 (35.28, 139.65)^**^	c': 1.17 (0.28, 2.06)^**^ b: 0.17 (0.07, 0.27)^**^	0.15^*^	1.17^**^	1.32^**^	11.1%
Choice reaction times	c: 1.32 (0.43, 2.20)^**^	a: −0.01 (−0.02, −0.001)^*^	c': 1.21 (0.32, 2.09)^**^ b: −9.53 (−14.56, −4.50)^**^	0.11	0.21^**^	1.32^**^	8.3%
Sit and reach	c: 1.32 (0.43, 2.20)^**^	a: 1.69 (0.80, 2.58)^**^	c': 1.02 (0.14, 1.90)^*^ b: 0.18 (0.12, 0.23)^**^	0.30^**^	1.02^*^	1.32^**^	22.5%
Grip strength	c: 1.32 (0.43, 2.20)^**^	a: 0.41 (−0.06, 0.89)	c': 1.21 (0.33, 2.09)^**^ b: 0.26 (0.15, 0.37)^**^	0.11	1.21^**^	1.32^**^	8.1%
Five times sit-to-stand	c: 1.32 (0.43, 2.20)^**^	a: −0.47 (−0.67, −0.27)	c': 0.98 (0.09, 1.86)^*^ b: −0.72 (−0.98, −0.46)^**^	0.34^**^	0.98^*^	1.32^**^	25.8%
BMI	c: 1.32 (0.43, 2.20)^**^	a: −1.09 (−1.38, −0.80)^**^	c': 0.81 (−0.09, 1.71) b: −0.47 (−0.64, −0.29)^**^	0.51^**^	0.81	1.32^**^	38.4%

FOF, fear of falling; PF, physical fitness; PA, physical activity (MVPA time; increased per 30 min/day); ^*^ and ^**^ indicate significance at 0.05 and 0.01 levels, respectively.

**Figure 1:**
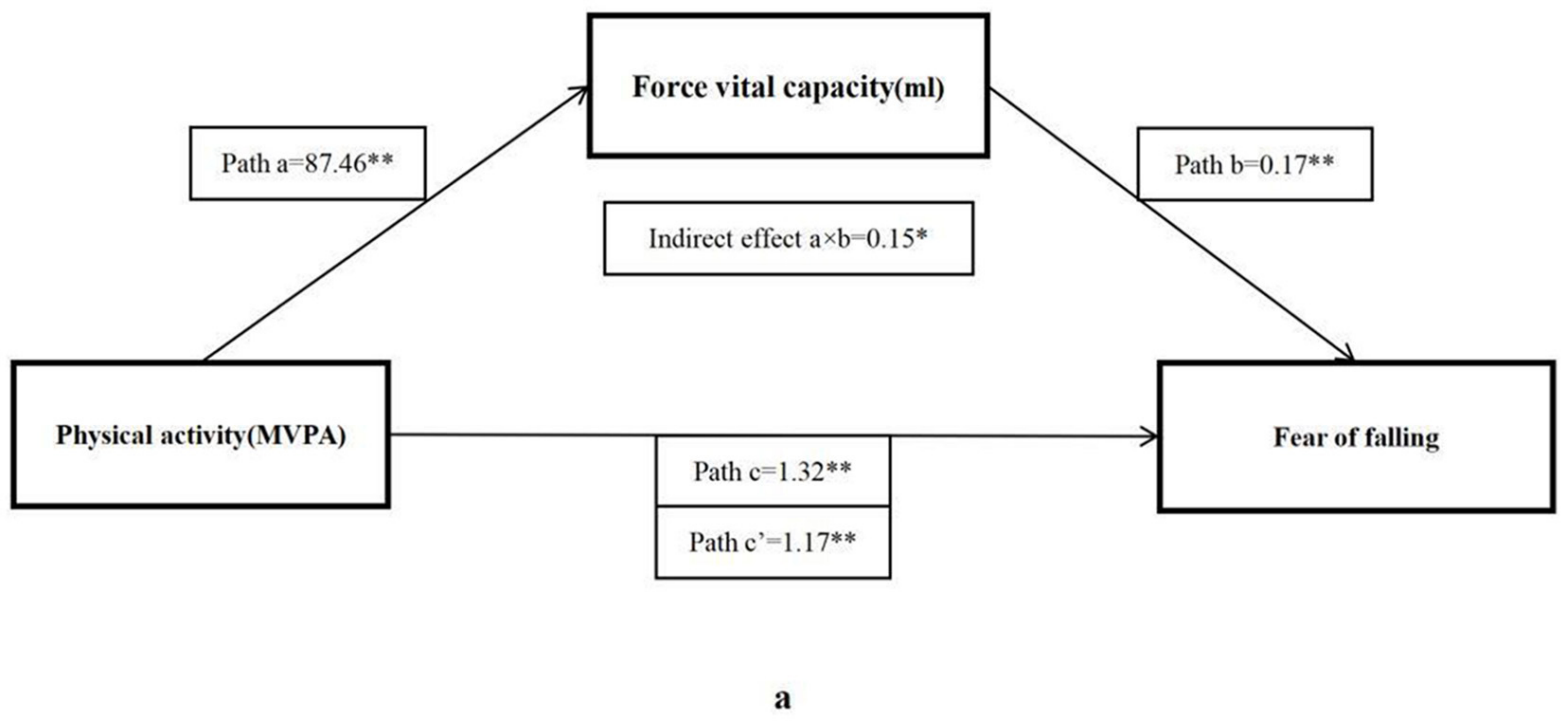
The mediating role of forced vital capacity in the association physical activity and fear of falling. The ^*^ and ^**^ symbols indicate significance at 0.05 and 0.01 levels, respectively.

**Figure 2:**
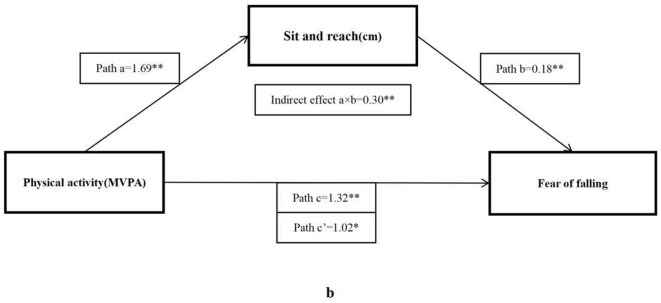
The mediating role of sit and reach in the association physical activity and fear of falling. The ^*^ and ^**^ symbols indicate significance at 0.05 and 0.01 levels, respectively.

**Figure 3:**
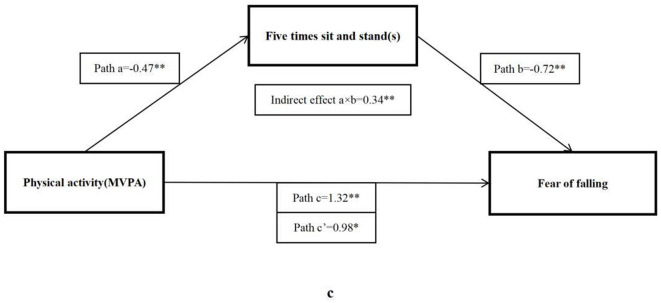
The mediating role of five times sit-to-stand in the association physical activity and fear of falling. The ^*^ and ^**^ symbols indicate significance at 0.05 and 0.01 levels, respectively.

**Figure 4:**
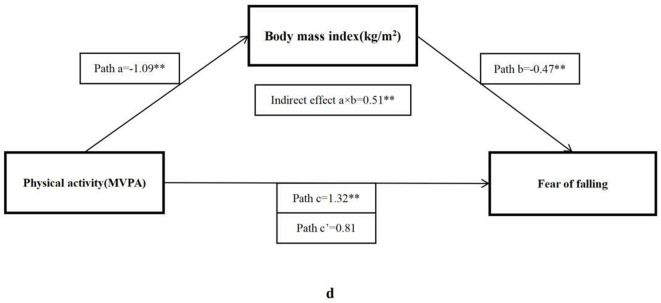
The mediating role of BMI in the association physical activity and fear of falling. The ^*^ and ^**^ symbols indicate significance at 0.05 and 0.01 levels, respectively.

The bootstrap analysis verified the mediating effect (Table 5). We also found that physical fitness plays an important role in the relationship between MVPA and FOF. The results suggested that forced vital capacity, sit and reach, five times sit-to-stand, and BMI were independent mediators of the effect of MVPA on FOF, and their mediating effect accounted for 11.1, 22.5, 25.8, and 38.4% of the total effect, respectively. Figures 1–4 present the mediation model used to determine whether PF indicators mediate the effect of MVPA on FOF.

**Table 5:** Bootstrap analysis.

**Mediator factors**	**Effect**	**Effect size**	***p***	**Boot SE**	**Bootstrap 95% CI**	
					**Lower limit**	**Upper limit**
Forced vital capacity	Indirect effect	0.146	0.019	0.063	0.024	0.269
	Direct effect	1.170	0.011	0.463	0.263	2.077
Choice reaction time	Indirect effect	0.110	0.065	0.060	−0.007	0.228
	Direct effect	1.206	0.010	0.466	0.292	2.120
Sit and reach	Indirect effect	0.297	0.003	0.101	0.099	0.494
	Direct effect	1.020	0.026	0.457	0.125	1.915
BMI	Indirect effect	0.507	0.005	0.180	0.154	0.860
	Direct effect	0.810	0.054	0.421	−0.015	1.634
Five times sit-to-stand	Indirect effect	0.339	0.003	0.113	0.118	0.561
	Direct effect	0.977	0.025	0.435	0.125	1.830
Grip strength	Indirect effect	0.107	0.098	0.065	−0.020	0.233
	Direct effect	1.210	0.009	0.464	0.301	2.119

Boot standard error (Boot SE); bootstrap 95% confidence interval (Bootstrap 95% CI).

## Discussion

This cross-sectional study aimed to determine whether a mediating role by PF exists in the relationship between PA and FOF in community-dwelling older women. The main findings of this study were that MVPA was significantly and negatively correlated with FOF while LPA did not. In addition, a significant association was found between MVPA, BMI, forced vital capacity, choice reaction time, sit and reach, grip strength, five times sit-to-stand, and FOF. The benefits of MVPA on FOF are fully mediated by BMI and partially mediated by forced vital capacity, sit and reach, and five times sit-to-stand performance. These findings provide a deeper insight into the mechanism of PA on FOF.

Both regular PA and health-related physical fitness are key indicators of health (20, 21). A positive association between PA and PF has been previously reported (28, 48). PA can help to maintain a healthy weight status and improve aerobic fitness, muscular fitness, and body composition (28, 49–51). These effects seem to depend on the intensity and volume of PA, and the results were not always consistent (48). Our study was consistent with most previous studies that higher MVPA was positively correlated with lower BMI, higher grip strength, and better sit and reach performance in older women. However, no significant correlation between MVPA and sit and reach was identified in older adults (28). In addition, our study also suggested that MVPA was correlated with shorter reaction time and shorter time to complete five times sit-to-stand, which was not elucidated in older women by previous studies. This can be explained by the evidence that MVPA usually involves more strenuous and faster muscle contractions, thus increasing muscle strength or power (52).

This study also suggested that MVPA rather than LPA was significantly correlated with ABC score; hence, a negative correlation was observed with FOF. The logistic regression results showed that the odds of high FOF significantly declined by 28% with each 30 min increase in daily MVPA. This finding is in line with the study led by Whipple et al. who found that participants with less MVPA tended to report higher FOF; however, only 10 older adults were enrolled in the study (53). Moreover, our previous study also demonstrated that older women in higher tertile of daily MVPA (>38.3 min) had a lower risk of high FOF (19). A systematic review summarized the relationship between PA and FOF in older adults and concluded that higher PA including total PA and MVPA was associated with lower FOF (54). Apparently, the accumulation of more daily MVPA time was associated with the improvement of FOF in older women. However, our study found that the increase in LPA was not associated with FOF. In the same samples, our previous study suggested that even the highest tertile of LPA (>336.4 min per day) was not associated with FOF (19). Previous studies consistently suggested a significant negative association between FOF and LPA in older adults after controlling confounding factors (17, 55). The difference might be due to the definition of LPA which was defined as 1.1–2.9 METs, the participants having an average of older age, and these studies enrolling participants with both genders.

This study confirmed the mediating role of PF in the relationship between MVPA and FOF. Older women with more daily MVPA had a lower FOF risk, and this was largely mediated by their better PF. This finding is consistent with the idea that PF is a strong predictor of the overall health of older adults (56) and contributes to better balance confidence (57) or lower concern of falling (58), hence a low risk of fall (59). MVPA is one of the primary modifiable factors capable of influencing PF of the old population (28, 60, 61), while higher PF level can influence fall efficacy or balance confidence to reduce FOF in older adults. A Korean study found that most physical fitness factors including 6-min walk, 30-s chair stand, 30-s arm curl, sit and reach, and grip strength were strongly associated with decreased FOF (62). This suggested that PF played an important role in reducing FOF. The logistic regression analysis indicated a significant association between BMI and FOF. This finding is consistent with previous studies that FOF is high in obese subjects (63). People with higher BMI will restrict physical activity, regardless of whether they have falling experiences (63). The mediating effect of BMI accounted for 38.4% of the total effect of PF which reminded us of maintaining an appropriate BMI to reduce the risk of FOF.

Muscular fitness is also an important indicator of physical health in the old population. Many studies have explored the relationship between muscle strength and FOF, and the results seemed inconsistent due to the usage of different muscle test tools (64–67). In this study, we did not find a mediating role in upper limb strength. Although no mediating effect was found, the linear relationship between grip strength and FOF is significant. Thus, we cannot deny the benefits of better upper limb strength on FOF. This finding is consistent with the previous results that older adults with FOF have lower grip strength compared with those without FOF (65), and the grip strength level of women with FOF is lower than that of men with FOF (68). Moreover, the five times sit-to-stand, a commonly used measure of sit-to-stand performance in older adults, is associated with dynamic balance. The time to complete the five times sit-to-stand test is significantly longer in older individuals with balance dysfunction than in healthy control individuals (69). This study demonstrated that the time to complete five times sit-to-stand was significantly associated with FOF. This observation was in line with a recent study in adults aged 50 years and older (70). Sit and reach was widely used to evaluate flexibility, which is associated with improving joint range of motion and function, and is important for the daily activity performance in older adults. Only a few studies explored the association between flexibility and health outcomes, including arterial stiffening (71, 72) and the onset of atherosclerosis (73). For the first time, this study found that older women with high FOF had worse sit and reach performance and confirmed a mediating role of flexibility in the relationship between MVPA and FOF. This means that the accumulation of more MVPA partially reduces FOF by improving lower limb flexibility. Finally, we found that forced vital capacity is also an independent mediator in the relationship between MVPA and FOF. It is highly rational that there is a significant correlation between strenuous MVPA and forced vital capacity, which was found associated with functional performance in older adults (42, 74, 75). However, so far, no study has directly investigated the association between forced vital capacity and FOF in older adults. More relevant research is needed in the future to explore the role of physical fitness in FOF management.

## Conclusion

The findings of this study confirmed our hypothesis that physical fitness mediated the association between PA and FOF in older women. Specifically, an accumulation of more daily MVPA was associated with a reduced risk of FOF rather than LPA. The association between MVPA and FOF was fully mediated by BMI and partially mediated by sit and reach, five times sit-to-stand, and forced vital capacity. These findings highlight the important role of PF in the relationship between PA and FOF, which deepens our understanding of the impact of PA on FOF in older women. Therefore, developing interventions to promote MVPA participation is urgently needed to reduce FOF in older women.

## Limitations

There are several limitations to this study. First, the cross-sectional design limited the ability to establish a causal link, and longitudinal studies are needed in the future. Second, the participants recruited in this study were not nationally representative of the whole older population, future studies need to include older men as well, and a multi-center study is needed to better reflect the physical activity and fear of falling situation in the whole Chinese older population.

## Data availability statement

The raw data supporting the conclusions of this article will be made available by the authors, without undue reservation.

## Ethics statement

The studies involving humans were approved by Ethics Committee of the School of Nursing and Rehabilitation, Shandong University, China (2021-R-001). The studies were conducted in accordance with the local legislation and institutional requirements. The participants provided their written informed consent to participate in this study. No potentially identifiable images or data are presented in this study.

## Author contributions

SW: data curation, formal analysis, visualization, and writing—original draft preparation. GL: formal analysis, visualization, and writing—reviewing and editing. BS: data curation and formal analysis. SW and HG: methodology and writing—review and and supervision. QH: conceptualization, writing—reviewing editing, project administration, and funding acquisition. All authors have read and agreed to the submitted version of the manuscript.
